# Authors’ reply

**DOI:** 10.4103/1817-1737.69124

**Published:** 2010

**Authors:** Aiman El-Saed, Badriah Al-Otaibi, Hanan H Balkhy

**Affiliations:** 1 *Department of Infection Prevention and Control, King Abdulaziz Medical City, Riyadh; Gulf Cooperation Council (GCC), States and WHO Collaborating Center for Infection Prevention and Control, Saudi Arabia*; 2 *Department of Infection Prevention and Control, King Abdulaziz Medical City, Riyadh; Gulf Cooperation Council (GCC), States and WHO Collaborating Center for Infection Prevention and Control, Saudi Arabia. E-mail: balkhyh@hotmail.com*

Sir,

We thank Dr. Wiwanitkit[1] for his interest in our recently published article about our experience in improving rates of influenza vaccination coverage among healthcare workers (HCWs) at our tertiary care facility in Riyadh, Saudi Arabia.[2] In this article, training advocate nurses together with engaging nursing service in the efforts to enhance the vaccination coverage may have contributed to about 2.5 folds increase of vaccination coverage (from 29 to 77%). We believe we still have a room for further improvement especially after the slight drop happened last year concomitant with the recent H1N1 influenza pandemic (April 2009).

Dr. Wiwanitkit raised important questions about the best techniques to improve influenza vaccination coverage among HCWs.[1] We believe such improvement requires enhancement at all levels of any successful influenza vaccination program including education, training, engagement, motivation, accessibility, and mandate.[3,4] Although we carried out huge efforts at all these levels, careful evaluation of 2009–2010 influenza vaccination campaign revealed more to be done to achieve our goal of more than 90% coverage. In addition to the overlapping educational and advertising materials used, we are planning to start 2010–2011 campaign by adding online learning module for all HCWs and online quiz for advocate nurses to improve influenza vaccination education and training. These new materials will challenge the myths that were linked to H1N1 vaccine and negatively impacted the last influenza vaccination campaign. We are planning to continue the fruitful partnership with nursing service that was shown to be successful in our facility and elsewhere.[2,5] Free-vaccine accessibility will be further improved by adding weekend sessions at the employee health clinic.

Although we mandated influenza vaccination in 2009–2010 campaign in response to the recent H1N1 influenza pandemic, we did not get better rates of influenza vaccination coverage. We are planning a new modification in implementing the vaccination mandate in order to achieve the coverage goal. The signed waiver that was used inappropriately by some HCWs to report health-related reasons for decline needs to be replaced by an infection control “granted” waiver after reviewing a standard waiver application and supporting medical documentations. In case of waiver denial by the infection control staff, the HCW will have to get the vaccine or wear surgical mask while at work throughout the flu season, to maintain a fitness-for-duty requirement to continue working. Such approach was recently shown to be extremely successful in sustaining influenza vaccination coverage at 98% for many years.[6] In conclusion, no single technique is able to achieve high influenza vaccination coverage in HCWs, rather, a multipronged approach is required to achieve and sustain such a goal. Needless to say, without a strong support and commitment of the facility leadership, such approach would be difficult to implement.
